# Intraspecific morphological variation of *Bellidiastrum michelii* (Asteraceae) along a 1,155 m elevation gradient in the Tatra Mountains

**DOI:** 10.7717/peerj.11286

**Published:** 2021-04-16

**Authors:** Piotr Kiełtyk

**Affiliations:** Institute of Biological Sciences, Cardinal Stefan Wyszynski University in Warsaw, Warszawa, Poland

**Keywords:** Altitudinal gradient, Biomass, Floral characteristic, Within-species variation

## Abstract

Plant species that inhabit large elevation gradients in mountain regions are exposed to different environmental conditions. These different conditions may influence plant morphology via plastic responses and/or via genetic adaptation to the local environment. In this study, morphological variation was examined for *Bellidiastrum michelii* Cass. (Asteraceae) plants growing along a 1,155 m elevation gradient in the Tatra Mountains in Central Europe. The aim was to contribute to gaining a better understanding of within-species morphological variation in a mountain species across elevation gradients. Twelve morphological traits, which were measured for 340 plants collected from 34 sites, were plotted against elevation using Generalised Additive Models. Significant variation in *B. michelii* morphology was found across the elevation gradient. Plant size, in the form of plant height, total aboveground mass and total leaf mass, decreased significantly with increasing elevation. Similarly, floral traits, such as flower head mass, total flower mass, individual flower mass, flower head diameter and ligulate and tubular flower length, also decreased significantly with increasing elevation. However, the changes in these floral traits were not as large as those observed for plant size traits. Interestingly, the number of flowers produced by the plant, both ligulate and tubular, did not change across the studied elevation gradient. In this study, elevation was found to be an important gradient across which significant intraspecific morphological variation occurred in a mountain plant. These morphological changes may have occurred in response to various abiotic and biotic factors that change along elevation gradients.

## Introduction

Plants that grow across mountain slopes experience different stresses caused by the variable environmental conditions found along elevation gradients. The most notable changes associated with increasing elevation in mountain regions include decreases in the mean air temperature, shortening of the growing season, increases in light intensity, UV radiation and wind velocity, reductions in carbon dioxide and oxygen concentrations, and decreases in nutrient availability (Billings, 1974; Körner, 2003; Nagy & Grabherr, 2009). Soil microbial activity and resource competition intensity also decrease at higher elevations (Körner, 2003). Meanwhile, competition for pollinator services increases with elevation as the number of pollinators declines at higher elevations (Maad, Armbruster & Fenster, 2013; Zhao & Wang, 2015; Arroyo, Pacheco & Dudley, 2017). Elevation therefore encompasses many abiotic and biotic components. Thus, elevation likely acts as a key summary variable that relates to the phenotypic variation observed in plants growing on mountains (Kelly, 1998; Hautier et al., 2009; Scheepens & Stöcklin, 2013; He et al., 2017; Miljković et al., 2019; Paudel et al., 2019).

Plants growing along large elevational ranges can adapt to the local conditions at the different elevations by adjusting their morphology and allocation of biomass to different vegetative and reproductive structures (Guo et al., 2012; Takahashi & Matsuki, 2016). For example, plant height is a trait that is related to fitness through vegetative competitiveness and ability to use available resources. Plant height has commonly been found to decrease within species with increasing elevation (Nishizawa et al., 2001; Alexander et al., 2009; Zhu et al., 2010; Maad, Armbruster & Fenster, 2013; He et al., 2017; Paudel et al., 2019), possibly as a response to limiting climatic conditions (Körner, 2003). Moreover, flowers, which are organs that are relatively invariant within species, have been found to vary quantitatively in plants of the same species across mountain elevation gradients (Herrera, 2005; Maad, Armbruster & Fenster, 2013; Seguí et al., 2018). Intraspecific flower size can gradually increase (Kudo & Molau, 1999; Herrera, 2005; Maad, Armbruster & Fenster, 2013; He et al., 2017) or steadily decrease (Totland, 2001; Zhao & Wang, 2015; Hattori et al., 2016) with increasing elevation. Increased intraspecific flower size at high elevations, as observed in some entomophilous species, has been explained by pollinator selection for larger flowers at high elevations. This is because, at high elevations, pollinators are rare but generally have a greater size (Malo & Baonza, 2002; Maad, Armbruster & Fenster, 2013). At high elevations, the reproductive success of outcrossing insect-pollinated plants is expected to be limited by pollination (Zhao & Wang, 2015; Theobald, Gabrielyan & HilleRisLambers, 2016; Arroyo, Pacheco & Dudley, 2017). An increased flower size corresponds to an increased insect visitation rate (Totland, 2004), which, in turn, increases the chances of producing viable seeds and achieving reproductive success (Arroyo, Primack & Armesto, 1982; Ohara & Higashi, 1994; Bingham & Orthner, 1998). Conversely, reductions in flower size at high elevations may result from individual plastic responses to climatic constraints (Zhao & Wang, 2015). Flower size reductions may also result from local adaptation via abiotic selection for smaller flowers in unfavourable environmental conditions. This selection occurs due to the lower development and maintenance costs associated with smaller flowers (Herrera, 2009).

Different selective abiotic and biotic pressures occurring at different elevations (Frei et al., 2014) may further generate non-linear responses in plant morphological traits along elevation gradients (Malo & Baonza, 2002). Such non-linear responses have been reported for floral traits; a unimodal relationship was found between flower size and elevation, with the maximum flower size of *Cytisus scoparius* (L.) Link (Malo & Baonza, 2002), *Solidago minuta* L. (Kiełtyk, 2018) and *Viola maculata* Cav. (Seguí et al., 2018) occurring in the middle of the elevational range. Such unimodal relationships may result from the trade-off between pollinator selection for larger flowers (Totland, 2001; Totland, 2004; Malo & Baonza, 2002; Maad, Armbruster & Fenster, 2013) and climatic selection for flower miniaturisation at higher elevations (Herrera, 2009; Zhao & Wang, 2015).

Intraspecific morphological variation in plants growing across elevation gradients in mountain regions is gaining increasing research attention (e.g.,  Takahashi & Matsuki, 2016; He et al., 2017; Olejniczak et al., 2018; Seguí et al., 2018). Knowledge of this variation can help us to understand how plants have adapted to different environmental conditions found across steep mountain gradients. In turn, this allows us to predict possible plant responses to climatic change, particularly in cold mountain environments (Theurillat & Guisan, 2001; Frei, Bodin & Walther, 2010). In this study, phenotypic variation was investigated in the high-mountain plant, *Bellidiastrum michelii* Cass. (Asteraceae), across a 1,155 m elevation gradient in the Tatra Mountains, Central Europe. The variation in *B. michelii* traits with elevation were assessed using Generalized Additive Models (GAMs). GAMs provide flexible, nonlinear functions that can account for both linear and non-linear phenotypic responses without the need for *a priori* selection of candidate models. The aim of this study was to contribute to obtaining a better understanding of morphological variation in a single mountain species across elevation gradient. Specifically, the following questions were addressed: do morphological traits of *B. michelii* significantly change across the elevation gradient? And if so, what is it the variation pattern of these traits in relationship to the elevation gradient?

## Material and Methods

### Species and study area

**Figure 1:**
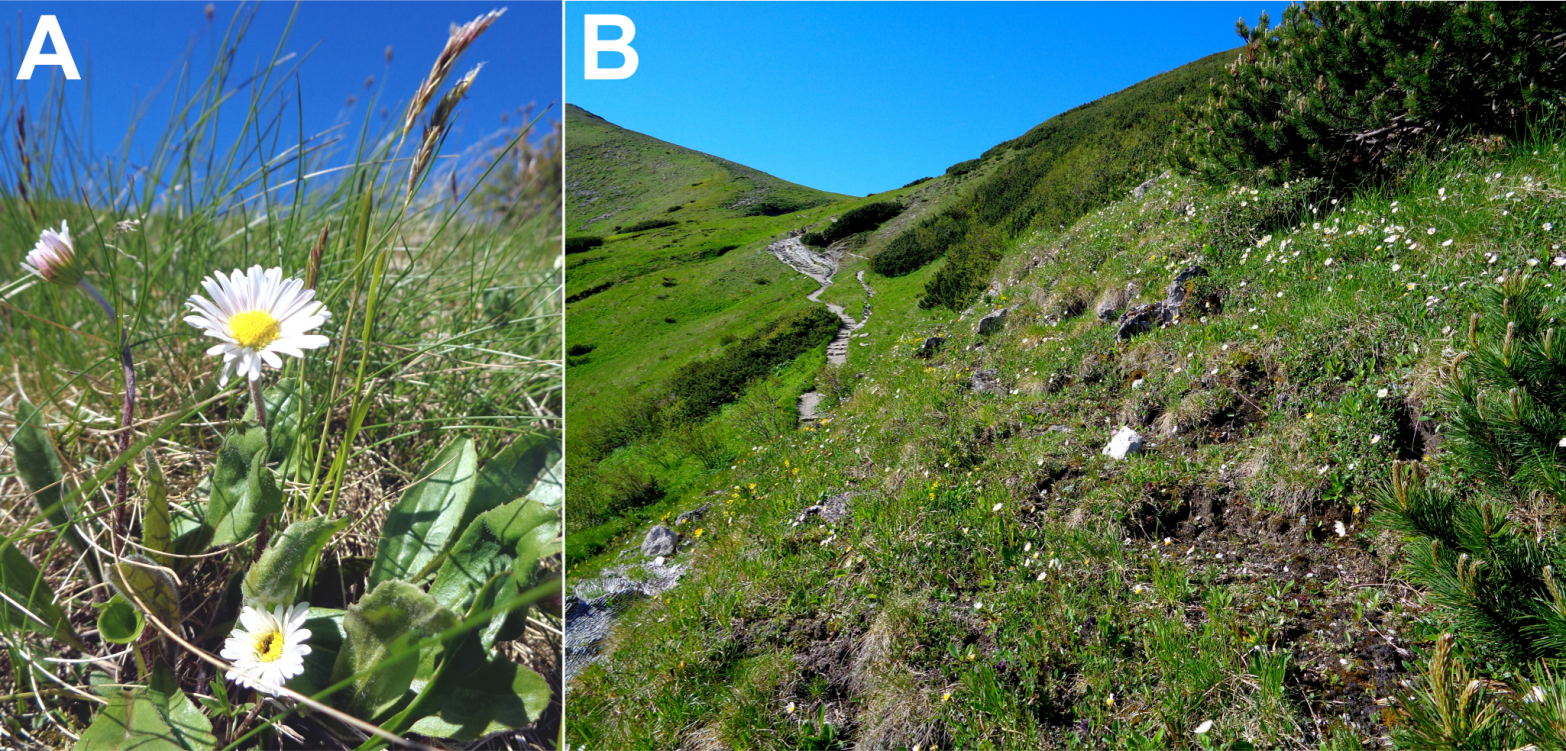
*Bellidiastrum michelii* plants (A) and the species habitat (B) with white flowering *B. michelii* plants in the subalpine belt at an elevation of 1,700 m a.s.l. in the Tatra Mountains.

*Bellidiastrum michelii* Cass. (Asteraceae, syn. *Arnica bellidiastrum* All., *Aster bellidiastrum* (L.) Scop., *Doronicum bellidiastrum* L.) is a perennial plant growing in the mountains of Central and Southern Europe, from the Western Carpathians, Alps and Jura to the Apennines and the western part of the Balkan Peninsula (Merxmüller & Schreiber, 1976; Aeschimann et al., 2004). The scape of *B. michelii* is erect, 10–35 cm long, not leafy, with one flower head at the top (Fig. 1). Leaves are suborbicular, spatulate, obovate or elliptical in shape and gathered in a basal rosette. Flowers, gathered in the flower head, are insect-pollinated, with outer female white or pink ligulate flowers and inner hermaphrodite yellow flowers. The species blooms from May to August (Rostański, 1971; Merxmüller & Schreiber, 1976). *B. michelii* grows predominantly in grasslands, pastures, on rocks and along springs on calcareous substrate (Rostański, 1971; Aeschimann et al., 2004).

**Figure 2:**
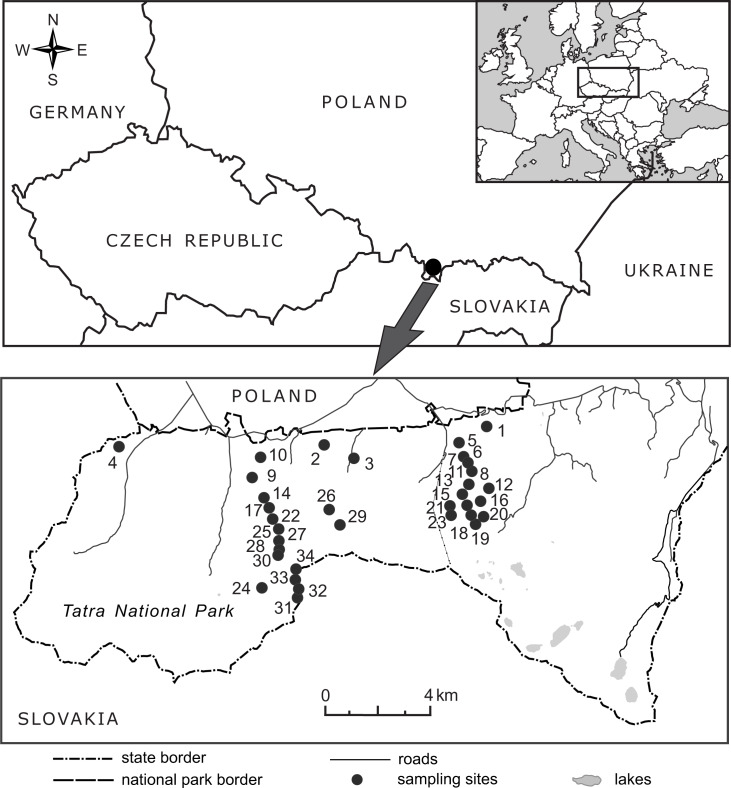
Location of the 34 elevational sites sampled for *Bellidiastrum michelii* in the Polish Tatra Mountains. For geographic coordinates and elevations, see Table 1.

The research was conducted along an elevation gradient in the Tatra Mountains in southern Poland (Fig. 2), within the protected area of the Tatra National Park (study permission of the Tatra National Park: Bot/380 DBN.503/63/16). The Tatras have the highest altitudinal range among Carpathian Mountains (the highest peak, Gerlach, is at 2,655 m a.s.l. in the Slovak part of the mountains), with a well-developed subnival zone at the highest elevations. The general elevational range in the Polish Tatras extends from ca. 900 m to 2,500 m a.s.l; the lower montane belt extends from an elevation of 600 m a.s.l. in the lower regions of the Carpathian Mountains and reaches an elevation of 1,250 m a.s.l. in the Tatra Mountains. The upper montane belt extends from ca. 1,250–1,500 (1,550) m a.s.l., the subalpine belt from ca. 1,500 (1,550)–1,800 m a.s.l., the alpine belt from ca. 1,800–2,300 m a.s.l. and the subnival belt above 2,300 m a.s.l. (Mirek & Piekoś-Mirkowa, 1992). In the literature, *B. michelii* has been reported in the Polish Tatras at elevations ranging from ca. 800 m a.s.l. in the Tatra foothills to 2,150 m a.s.l. (Rostański, 1971).

### Field sampling and morphological measurements

In 2016, from the end of May to the beginning of July, the flowering plants of *B. michelii* were collected from 34 sites ranging from 920 m a.s.l. to 2,075 m a.s.l. (Fig. 2, Table 1). An attempt was made to ensure that the sampled sites were distributed approximately evenly along the elevational range of the species (Table 1). The elevation at each site was determined in the field using two devices: a GPS receiver with built-in barometric altimeter (Garmin GPS MAP 76s, Olathe, USA) and a wrist barometric altimeter (Suunto Core All Black, Vantaa, Finland); obtained elevation records were also consulted. To assure recording accuracy, the altimeters of both devices were frequently calibrated at points of known elevation, as read from topographic maps and field elevation markers. The geographic coordinates and elevations of the study sites are presented in Table 1.

**Table 1:** Study sites of *Bellidiastrum michelii* in the Polish Tatra Mountains. Geographic coordinates were determined with a WGS84 geodetic system.

Site	Elevation (m a.s.l.)	Latitude (N)	Longitude (E)	Date
1	920	49°16′53.8″	19°59′53.3″	2016-05-30
2	955	49°16′23.1″	19°55′01.5″	2016-06-17
3	970	49°16′08.8″	19°55′50.5″	2016-06-19
4	1040	49°16′36.0″	19°49′31.2″	2016-05-28
5	1090	49°15′43.6″	19°59′05.6″	2016-06-17
6	1135	49°15′29.9″	19°59′25.9″	2016-06-20
7	1162	49°15′28.8″	19°59′32.3″	2016-06-17
8	1225	49°15′25.5″	19°59′37.0″	2016-06-20
9	1253	49°15′12.7″	19°52′52.5″	2016-06-16
10	1290	49°16′00.3″	19°53′23.4″	2016-05-27
11	1305	49°15′16.1″	19°59′40.5″	2016-06-20
12	1330	49°15′41.9″	19°59′42.0″	2016-06-20
13	1370	49°15′15.3″	19°59′47.4″	2016-06-20
14	1385	49°15′02.4″	19°53′05.9″	2016-06-16
15	1400	49°15′09.1″	19°59′52.6″	2016-05-29
16	1430	49°15′17.4″	19°59′57.7″	2016-06-20
17	1470	49°14′55.0″	19°53′12.0″	2016-06-16
18	1480	49°15′15.9″	20°00′08.3″	2016-06-20
19	1505	49°15′16.3″	20°00′13.2″	2016-06-20
20	1531	49°15′21.0″	20°00′16.9″	2016-06-20
21	1570	49°14′56.6″	19°59′49.2″	2016-06-19
22	1600	49°14′41.5″	19°53′15.8″	2016-06-21
23	1620	49°14′52.8″	19°59′49.5″	2016-06-19
24	1660	49°13′36.6″	19°53′52.6″	2016-06-18
25	1695	49°14′34.0″	19°53′25.2″	2016-06-21
26	1707	49°14′38.7″	19°54′42.2″	2016-07-04
27	1755	49°14′26.0″	19°53′28.2″	2016-06-21
28	1790	49°14′20.1″	19°53′32.1″	2016-06-21
29	1818	49°14′41.4″	19°54′48.5″	2016-07-04
30	1843	49°14′18.7″	19°53′37.7″	2016-06-21
31	1925	49°13′38.4″	19°54′14.9″	2016-06-18
32	1977	49°13′42.0″	19°54′14.3″	2016-06-18
33	2030	49°13′46.9″	19°54′13.1″	2016-06-21
34	2075	49°13′49.6″	19°54′12.6″	2016-06-21

At each site, 10 plants with well-developed flower heads (in blossom peak) were sampled, and their aboveground parts (i.e., flowering scapes with leaf rosettes) were collected. Each of the sampled plants was sufficiently distant (>ca. 2 m) from other sampled plants to ensure they represented different genetic individuals. Sampled individuals were well-developed and did not show restriction in growth and reproductive function. In this study, 12 morphological traits were analysed; details on their measurements are presented in Table 2. The first measurement was the flower head diameter expressed as the maximum distance between the tips of two opposed petals. This measurement was made with a digital calliper (Powerfix Profi, Neckarlsum, Germany) immediately after a plant was collected, when the flowers were fresh and the flower head not deformed. The plants were then placed in a botanical press, between drying paper and jute fabric, and preserved for further analyses at the Plant Biology Laboratory of the Cardinal Stefan Wyszynski University in Warsaw. Plant height was assessed by measuring the length from the plant base to the top of the flower head. The plants were further separated into three fractions (scape, leaves and flower head) and dried for 48 h at 80 °C in a laboratory drying oven with natural air circulation (Pol-Eko-Aparatura SLN 240, Wodzisław Śląski, Poland) to obtain the dry matter content (Pérez-Harguindeguy et al., 2013) by weighing on an analytical balance (Radwag AS 60/220.X2 PLUS, Radom, Poland). The flower heads were then soaked in water in a Petri dish for 1 h and then separated into ligulate and tubular flowers. This rehydration of the flower heads and flowers was done to enable the separation of the flower head without causing damage to the dry (and hence brittle) flowers and to ease straightening of the flower corollas for subsequent measurements. The flowers were counted and the width of the ligule and lengths of the ligulate and tubular flowers were measured using a stereoscopic microscope (Delta Optical SZ-450T, Mińsk Mazowiecki, Poland). The number of ligulate and tubular flowers was counted directly for each flower head, whereas the sizes of ligulate and tubular flowers were measured for one randomly sampled flower from the head. After the flowers were measured, they were stored in paper envelopes and dried for 48 h at 80 °C in a drying oven with forced air circulation (Binder FD 115, Tuttlingen, Germany) to obtain dry matter content, followed by weighing on the analytical balance. All weight measurements were carried out immediately after the samples were removed from the oven to prevent humidity absorption from air in the laboratory, which may influence the weight measurements.

**Table 2:** *Bellidiastrum michelii* traits used in the study.

Trait	Measurement details	Accuracy/significant digits
**Plant height**—measured from the scape base to the top of the flower head.	Measured with a ruler on herbarium specimens.	1 mm
**Plant mass**—dry mass of the aboveground plant parts.	Weighed on an analytical balance after drying for 48 h at 80 °C.	0.0001 g
**Total leaf mass**—total dry mass of all plant leaves.	As above.	0.0001 g
**Flower head mass**—dry mass of the flower head.	As above.	0.0001 g
**Total flower mass**—total dry mass of ligulate and tubular flowers in the flower head.	As above.	0.00001 g
**Individual flower mass**—calculated by dividing total flower mass in the flower head by total number of flowers in the head.	–	0.00001 g
**Flower head diameter**—the maximum distance between the tips of two opposed petals.	Measured with a digital calliper on fresh plants in the field immediately after plant collection.	0.1 mm
**Number of ligulate flowers** in the flower head.	Counted by stereomicroscopy at ×10 magnification after separation of the flower head into ligulate and tubular flowers.	–
**Number of tubular flowers** in the flower head.	As above.	–
**Ligulate flower length**—corolla length measured for one randomly chosen ligulate flower from the head.	Measured by stereomicroscopy at ×10 magnification after 1 h rehydration in water.	0.1 mm
**Ligulate flower width**—ligule width of the chosen flower.	As above.	0.1 mm
**Tubular flower length**—corolla length measured for one randomly chosen tubular flower from the head.	As above.	0.1 mm

### Statistical analyses

Statistical analyses were based on measurements of 12 morphological traits for 340 plants collected from 34 sites (10 plants from each site) distributed continuously along the 1,155 m elevation gradient. All statistical analyses were performed using R version 3.6.1 (R Core Team, 2019). Prior to the analyses, the normality of distribution was checked for the all studied variables by comparing the observed variable distribution with the theoretical normal distribution using the *ggqqplot()* function from the *ggpubr* package (Kassambara, 2019) and by conducting the Shapiro–Wilk test for normality with the *shapiro.test()* function of the *stats* package (R Core Team, 2019). None of the variables, with the exception of the number of ligulate flowers and the ligulate flower length, were normally distributed; therefore, to meet normal distribution, they were transformed with the Box–Cox method using the *boxcox()* function of the *bestNormalize* package (Peterson & Cavanaugh, 2019) and were standardised (mean = 0 and standard deviation = 1) for subsequent analyses. The performance of the various methods of data transformation available in the *bestNormalize* package was assessed and the Box–Cox method was found to produce the best data normalisation in terms of agreement with the theoretical normal distribution. However, for three variables (total flower mass in the head, tubular flower length and ligulate flower width), a normal distribution could not be met by any method of data transformation. Therefore, when assessing results of subsequent analyses for these three variables, caution is required. The shape of an elevational variation in morphological traits was assessed using Generalised Additive Models (GAMs). The GAMs were run on the transformed and standardised trait values, and the elevation variable was expressed in kilometres to avoid numerical estimation problems (Zuur, 2012). In the GAM models the elevation was treated as a fixed effect and sites of samples collection were set as a random model component. The GAMs were fitted with the Restricted Maximum Likelihood (REML) method. The analyses were accomplished with the *gamm4()* function available in the *gamm4* package (Wood & Scheipl, 2017). In the GAMs summary the effective degrees of freedom (edf) represent the complexity of the smoothing. An adf of 1 is equivalent to a straight line, an edf of 2 is equivalent to a quadratic curve, etc., with higher edfs describing more wiggly curves. The F-statistics and *P*-values are test statistics used in ANOVA to test the significance of the GAM models.

## Results

**Figure 3:**
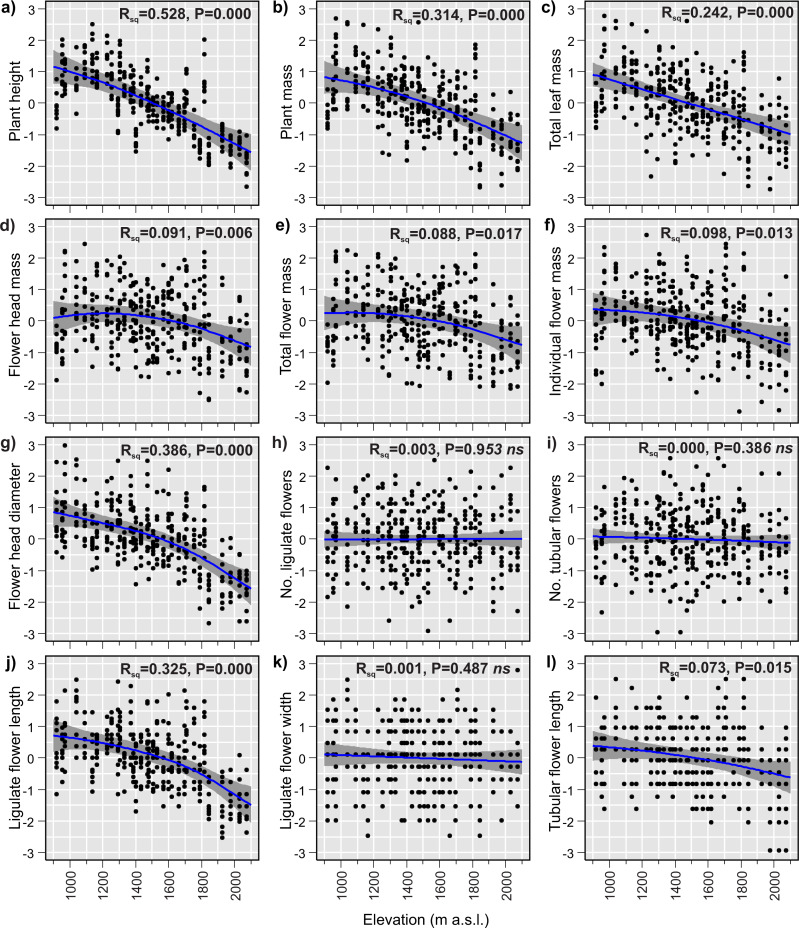
(A–I) Elevational variation in *Bellidiastrum michelii* traits fitted by Generalised Additive Models (GAMs). All traits are standardised (mean = 0 and standard deviation = 1); the grey band represents a 95% confidence interval for the mean shape of the effect (smoother). P –*p*-value of model significance test at 0.05 significance level; *ns*, non significant models. Summary of the GAMs are presented in Table 3.

The GAMs of the relationships between plant traits and elevation revealed that 9 of the 12 studied traits changed significantly with elevation (Table 3). Plant size decreased considerably with increasing elevation. The plant height, plant mass and total leaf mass decreased by 69%, 64% and 66%, respectively (Figs. 3A–3C), across the 1,155 m elevation gradient. Hereafter, the percentage changes in trait values will refer to the 1,155 m elevation gradient, unless stated otherwise. Flower head mass and total flower mass showed slightly hump-shaped patterns (Figs. 3D and 3E). Initially, from 920 m a.s.l. to 1,225 m a.s.l., the flower head mass increased by 3%, and then from 1,225 m a.s.l. to 2,075 m a.s.l, decreased by 22%. The total flower mass initially increased by 0.5% from 920 m a.s.l. to 1,040 m a.s.l., and then decreased by 23% from 1,040 m a.s.l. to 2,075 m a.s.l. However, it is important to note that these initial slight increases in flower head mass and total flower mass at low elevations may not be statistically significant. This is because the 95% confidence intervals for the fitted model lines were wider than the observed hump-shaped patterns in the fitted lines at low elevations (Figs. 3D and 3E). Meanwhile, the individual flower mass decreased by 20% (Fig. 3F) and the flower head diameter decreased by 25% (Fig. 3G) from the lowest to highest elevation. The number of flowers in the flower head, both ligulate and tubular, showed no elevational tendency. These traits were very variable at all sites across the elevational species range and did not exhibit any elevational trend (Figs. 3H and 3I). Ligulate flower length decreased by 23% between the lowest and highest elevation (Fig. 3J). Ligulate flower width showed high variation at all sites across the elevation gradient and did not exhibit any significant relationship with elevation (Fig. 3K). Tubular flower length decreased by 7% from the lowest to the highest elevation (Fig. 3L).

**Table 3:** Generalised Additive Model (GAM) summaries for fitting *Bellidiastrum michelii* traits to elevation. The significance level = 0.05; ns, non significant models. The effective degrees of freedom (edf) represent the complexity of the smoothing. An adf of 1 is equivalent to a straight line, an edf of 2 is equivalent to a quadratic curve, etc., with higher edfs describing more wiggly curves.

Trait	edf	F	P	R-sq (adj.)	Fitted value at 920 m a.s.l.	Fitted value at 2075 m a.s.l.	Change 920–2075 m a.s.l. (%)
Plant height (mm)	1.639	34.24	0.000	0.528	278.5	86.3	–69
Plant mass (g)	1.709	21.97	0.000	0.314	0.3152	0.1122	–64
Total leaf mass (g)	1.000	30.74	0.000	0.242	0.2040	0.0700	–66
Flower head mass (g)	2.198	4.93	0.006	0.091	0.0349	0.0279	–20^*^
Total flower mass (g)	1.870	5.54	0.017	0.088	0.0203	0.0157	–23^**^
Individual flower mass (g)	1.640	6.70	0.013	0.098	0.00015	0.00012	–20
Flower head diameter (mm)	2.256	27.21	0.000	0.386	29.93	22.56	–25
No. ligulate flowers	1.000	0.00	0.953 ***ns***	0.003	–	–	–
No. tubular flowers	1.108	0.90	0.386 ***ns***	0.000	–	–	–
Ligulate flower length (mm)	2.290	19.25	0.000	0.325	11.95	9.14	–23
Ligulate flower width (mm)	1.000	0.48	0.487 ***ns***	0.001	–	–	–
Tubular flower length (mm)	1.447	6.31	0.015	0.073	3.84	3.57	–7

**Notes.**

* Flower head mass had maximum fitted value of 0.0359 g at elevation 1,225 m a.s.l.

** Total flower mass had maximum fitted value of 0.0204 g at elevation 1,040 m a.s.l.

## Discussion

Most of the morphological traits of *B. michelii* varied significantly across the elevation gradient. The plant height, total aboveground mass and total leaf mass decreased considerably from low to high elevations. Similarly, floral traits, such as flower head mass, total flower mass, individual flower mass, flower head diameter, and ligulate and tubular flower length, also decreased with increases in elevation. However, the reductions in these floral traits were not as great as those observed for plant size traits. Meanwhile, the number of ligulate and tubular flowers produced by the plant did not change across the studied elevation gradient. The intraspecific variation observed in this study suggests that variable stress factors correlated with elevation impact plant morphotype. The elevational variation in morphology of *B. michelii* may result partially from phenotypic plasticity and partially from local adaptations at different elevations.

The considerable decrease in intraspecific plant size with increasing elevation observed in this study is in line with the results of previous studies (e.g., Nishizawa et al., 2001; Alexander et al., 2009; Zhu et al., 2010; Maad, Armbruster & Fenster, 2013; Paudel et al., 2019). These within-species size reductions may result from restricted growth caused by resource limitations, such as low air temperature, short growing seasons, strong winds, and shallow soil, that occur at high altitudes (Körner, 2003; Nagy & Grabherr, 2009). Moreover, these intraspecific plant size reductions may also partly result from local adaptation to high elevation environments via selection. Smaller phenotypes are thought to be more advantageous in limiting environmental conditions due to their lower resource requirements, and thus may be favoured by selection (Herrera, 2005; Zhao & Wang, 2015).

In this study, significant elevational variations were also found in several floral traits. In general, flowers are relatively invariant within a singular species, as this is important for securing the compatibility of the species mating system. Therefore, intraspecific variations in floral traits are often of adaptive significance (Nosil, 2012). Increasing flower size with increasing elevation has been found in several mountain species (e.g., Kudo & Molau, 1999; Maad, Armbruster & Fenster, 2013; He et al., 2017). This increase in flower size is thought to represent an adaptation that enhances flower attractiveness to pollinators (Herrera, 2005; Maad, Armbruster & Fenster, 2013). However, in *B. michelii*, neither flower nor flower head size increased with increasing elevation. On the contrary, the sizes and masses of the flowers and flower heads of *B. michelii* decreased as elevation increased. These observed reductions in the floral traits of *B. michelii* at high elevations are in line with the results of other studies that found that within-species flower size decreased with increasing elevation (e.g., Totland, 2001; Zhao & Wang, 2015; Hattori et al., 2016). Reductions in flower size and mass with increasing elevation can be linked to the more limiting conditions caused by increases in climate severity at high altitudes. Intraspecific flower miniaturisation has been suggested to be advantageous for plants growing under the resource-limited environmental conditions of high mountain elevations, due to the lower cost of flower development and maintenance (Herrera, 2009; Zhao & Wang, 2015).

In conclusion, significant intraspecific morphological variations were found in *B. michelii* across the elevation gradient. The results of this study suggest that important ecological factors associated with elevation gradients drive intraspecific morphological variation in mountain plants growing across large elevational ranges. It will be possible to identify these drivers by thoroughly analysing local abiotic (such as climatic) and biotic (such as pollinator services) ecological factors across elevation gradients. Notably, the observed elevational variation in *B. michelii* probably results partially from plastic responses to abiotic (mainly climatic) conditions and partially from genetic adaptation to locally prevailing conditions via selection. However, further study will be required to disentangle the contributions of genetic differences among locally adapted populations, and of plastic responses, to morphological variation across elevation gradients. Such further study will need to be based on experiments involving reciprocal plant transplantation to different mountain elevations (e.g., Gonzalo-Turpin & Hazard, 2009; Hautier et al., 2009; Scheepens, Frei & Stöcklin, 2010; Hamann et al., 2016).

##  Supplemental Information

10.7717/peerj.11286/supp-1 File S1 Raw data for Intraspecific morphological variation of *Bellidiastrum michelii* (Asteraceae) along a 1,155 m elevation gradient in the Tatra Mountains.

## Additional Information and Declarations

### Field Study Permissions

The following information was supplied relating to field study approvals (i.e., approving body and any reference numbers): Field experiments were approved by the Tatra National Park (permission Bot/380, DBN.503/63/16).

### Data Availability

The following information was supplied regarding data availability: The raw measurements are available as a Supplemental File.
